# Classification, Perception, and Toxicity of Emerging Flavored Oral Nicotine Pouches

**DOI:** 10.3390/ijerph20054526

**Published:** 2023-03-03

**Authors:** Sadiya Bi Shaikh, Chad Newton, Wai Cheung Tung, Yehao Sun, Dongmei Li, Deborah Ossip, Irfan Rahman

**Affiliations:** 1 Department of Environmental Medicine, University of Rochester Medical Center, Rochester, NY 14642, USA; 2 College of Professional Studies, Bethel University, McKenzie, TN 38201, USA; 3 Department of Clinical and Translational Research, University of Rochester Medical Center, Rochester, NY 14642, USA; 4 Department of Public Health Sciences, University of Rochester Medical Center, Rochester, NY 14642, USA

**Keywords:** ONPs, flavors, perception, regulation, menthol, tobacco, nicotine, toxicity

## Abstract

Introduction: Oral Nicotine Pouches (ONPs) are the new form of nicotine pouches that have become a type of emerging smokeless tobacco product sold by various tobacco companies. These smokeless tobacco products are marketed for usage all over as snus containing tobacco-derived nicotine (natural) or as tobacco-free nicotine (synthetic) as substitutes for other tobacco products. Based on perception and socio-behavioral aspects, ONPs have become popular tobacco products among adolescents/young adults, and over 50% of young adult users of ONP use flavored ONPs, such as menthol/mint, tobacco, dessert/candy, and fruity, which are the most popular flavors. Various new ONP flavors are currently popular locally as well as in the online market. Tobacco, menthol, and fruit-flavored ONPs could motivate cigarette smokers to change to ONPs. Methods: We expanded our knowledge on natural/synthetic ONP flavor wheels to available data on ONPs, describing, in detail, their flavors and brands (US and Europe) in both natural and synthetic ONP categories. We classified over 152 snus and 228 synthetic ONPs into the following flavor categories: “Tobacco”, “Menthol/Mint”, “Fruity”, “Candy/Deserts”, “Drink”, “Aroma”, “Spices”, and “Mixed Flavors”. Results: Based on total numbers, we found the most popular ONP flavors, sold as tobacco and menthol, to be among natural ONPs; among synthetic ONPs, fruity and menthol are the most prominent flavors, with varying concentrations of nicotine and other flavoring chemicals, including coolant WS-23. We also showed possible molecular targets and toxicities, due to exposure to ONPs, activating several signaling cascades such as AKT and NF-kappaB, which might possibly lead to apoptosis and epithelial mesenchymal transition (EMT). Conclusions: Considering the marketing of ONP products with various flavor profiles and with most of these products containing tobacco/menthol/fruit flavor, it is likely to have regulation and a marketing disclaimer on some of these products. Further, it would be logical to determine how the market reacts in terms of compliance and non-compliance with flavor restrictions by the regulatory agencies.

## 1. Introduction

Oral Nicotine Pouches (ONPs) are a type of emerging smokeless tobacco product sold by some large tobacco companies [1]. Originating from Scandinavia, the selling of ONPs quickly spread to many other countries, including the European Union, the United Kingdom, the United States, and Japan [1,2,3]. The U.S. Food and Drug Administration (FDA) closely describes these oral pouches as “ground, cut, leaf or powdered tobacco regularly available in moist or chew gum snuff often packed in a pre-portioned pouch” [1,4]. The modern ONPs differ from traditional smokeless tobacco called snus, and these newer ONPs are tobacco-free with no tobacco leaf material. Instead, they are constructed of nicotine mined from tobacco leaf and marketed in various forms, including chewable tablets, nicotine pouches, gums, and lozenges [5,6]. The appearance of these tobacco-free pouches is similar to that of snus, and similarly, the ONPs are placed in a user’s anterior maxillary vestibule (between the gum and the lip) for chemicals to be released and absorbed [2,3]. Since 2016, the U.S. [2,7] market has consisted of nicotine pouches that do not contain tobacco leaves in the final product, and since 2018, they have been in Europe [2,8,9].

In spite of smokeless tobacco being prohibited in some countries, a relatively higher intake of these products has been reported in Sweden and the United States [10], as 1800 subjects with traditional usage of snus have been reported in Sweden [10]. Prime components of snus pouches noted are air or sun-cured tobacco, salt, water, and food-grade flavorings [6], whereas the ONPs are plant-based fibers boosted by flavorings, nicotine, and other ingredients [11,12]. In ONPs, the flavors are marketed as important components, as the availability of these flavors are believed to be the reason why ONPs attracted youth and adolescents, and are used broadly [13].

In the current scenario, the usage of ONPs serves as a gateway to quitting cigarette smoking. Referring to this fact, evidence states that the first Nicotine Replacement Therapy (NRT) was reported in the form of chewing gum, which was manifested to reduce or prevent symptoms from smoking abstinence [14,15]. NRTs are considered as short-term intervention with a motive to assist individuals to switch from cigarette smoking, substituting nicotine supplied by cigarettes [14]. In this context, a study of 133 trials on various NRTs stated that smokeless tobacco products such as ONPs, lozenges, chewing gums, sublingual tablets, nasal sprays, etc., exhibited a 50–60% increase in the rate of successful halting among smokers who are influenced to quit smoking [14,16]. However, the rate of successful cessation is not elevated for maximum smokers who just attempt the NRTs [12].

Since December 2020 in the United States, none of the oral nicotine products are regulated, as the FDA has not granted any authorization for ONPs to be sold as altered-risk tobacco products [17]. The FDA carries full authority to limit the usage of wrong or deceiving claims in the advertising of ONPs that may encourage users that intake of ONPs is harmless [18]. To prevent addiction and related health issues in individuals due to tobacco products, the FDA finalized the “Deeming Tobacco Products to Be Subject to the Federal Food, Drug, and Cosmetic Act” (the “Deeming Rule”) in 2016 [19]. This rule established the regulatory authority of the FDA on all tobacco products that include tobacco-derived nicotine (TDN), so manufacturers of these products need to have the products undergo pre-market assessments/authorizations, submit product information, and obey the restrictions [19,20]. However, the “Deeming Rule” did not include tobacco-free nicotine (TFN) products until the FDA amended the official definition of tobacco products into “any product made or derived from tobacco or containing nicotine from any source, that is intended for human consumption” to include TFN products [21]. As a result, ONPs are completely under the regulation of the FDA now, and all ONPs without a pre-market application/authorization (PMTA) will be removed from the market regardless of whether the nicotine content is TDN or TFN [19,21].

Over time, marketing of ONPs has increased substantially in the United States. As per reports, the commercial market share of ONPs in the United States jumped from 0.9% in 2018 to 4.0% in 2019 [22,23]. Reports also state that adolescents display high interest in the latest newer smokeless non-tobacco ONPs due to their resemblance with the preferred food products, such as chewing gums, and their accessibility in appealing flavors [13,22]. An illustrative check-over among adolescents and Dutch adults has recognized about 0.06% as present customers, and 0.56% as ever having been customers of ONPs [24]. In the United Kingdom (U.K.), among past and present smokers or e-cigarette users, 15.9% of participants were aware of ONPs, among which 2.7% were present users and 4.4% were ever consumers [25].

Various prime tobacco companies, including Altria, Swedish Match, and RJ Reynolds, are presently marketing ONPs and lozenges, offering flavors within various ranges of nicotine content [13,26]. There are various local vendors involved, similar to snus. and ONPs are also available in a multitude of flavors such as fruit, dessert, citrus, mint, coffee, berry, and wintergreen, which contributes to the prevalence of ONP utilization in the United States [27]. Modern non-tobacco ONPs are also involved in enrolling advertising and marketing perspectives, digital marketing campaigns, and marketing themes projecting minimal harm, especially in attracting the youth population [28].

### 1.1. Social Behavioral Aspects of Oral Nicotine Pouches

The sociological result of oral nicotine products, including vaping and pouches (smokeless products), through habitualization, commercialization, and normalization is a socially accepted illusion [29]. According to Berger and Luckmann (1966), a person’s sense of reality is socially constructed through human interactions, and those interactions involve repeated exposures and engagements with other habitual participants in the same or similar habit. As a result, a behavioral pattern or set of patterns becomes validated, thus starting a process called habitualization [30,31]. In a recent study, Clarke et al. (2021) stated that these products, including “e-cigarette devices and vaping fluids demonstrably contain a series of both definite and probable oncogenic responses by nicotine derivatives”. This includes benzo(a)pyrene and nitrosamines for oral, gastric, and liver effects [32]. Similar aspects can be attributed to flavoring compounds. However, this type of information does not usually reach public communities, especially where teenagers, adolescents, and other vulnerable populations congregate [22,33,34]. In fact, they use half-truths and deceiving statement—specifically, the message that harmlessness (non-toxic) of pouches/vaping exist in comparison to the effects of both cigarette/tobacco smoking [35]. Over a span of time, those half-truths and deliberate lies about addiction risks, as well as the physical effects of pouches/vaping and e-cigarettes, become realities among individuals and collective groups in society. Nitzkin (2014) emphasized the fact that “the tobacco-control movement is now the party deceiving the public through unfounded speculation and outright lies as to the risk posed by nicotine addictiveness to teen non-smokers (or withdrawal of nicotine polyproducts) due to perceptions behavioral effects” [22,33,34,35]. Therefore, the beginning of a socially accepted untruth became a firmly held norm. Once an untruth becomes normalized, a shared illusion develops and spreads through continued interactions containing the same or similar messages.

### 1.2. Commercialization, Perceptions, and Illusions of Nicotine Products

The commercialization of the illusion strengthens its influence through a constant buying-in, by consumers, to the false claims stated by producers and advocates of nicotine addiction through pouches and vaping. According to Boyer et al. (2020), “Vaping has been marketed as a safer alternative than smoking cigarettes, but safety data are lacking”, which can be extrapolated to ONPs [1,31]. In other words, inflated claims about the supposed innocence of nicotine influence the choices made by end users currently. As a result, demoralization occurs, in which realizations of untruths arouse moral outrage that breeds distrust among community members toward the organizations that participate in the spreading of deceptive or misleading messages about the use of these products through the deception of fruit and mint flavors [1,31]. For example, Boyer et al. (2020) emphasized the fact that “Addiction is central to the JUUL business model”. In other words, an intention to deceive candidates into becoming future nicotine users, and current ENDS users, exists and continues to influence public communities through marketing strategies and the de-emphasizing of harmful effects [31]. The most likely reason for the intention to deceive others in this way involves the profit motive, including the gateway to new users, by assuming that these products are not harmful.

Once an untruth becomes socially accepted and validated through positive reinforcement, the process of social normalization sets in, allowing the untruth behind the illusion to become truthful itself. At a point in time, the pattern of deception becomes embedded into the organizational culture of the companies that produce or derive benefits from advocating vape use, thus leading to further demoralization once the untruth is revealed and moral outrage erupts. Ashforth and Anand (2003) provided a pyramid that illustrated three aspects of organizational normalization [29].

**Figure 1:**
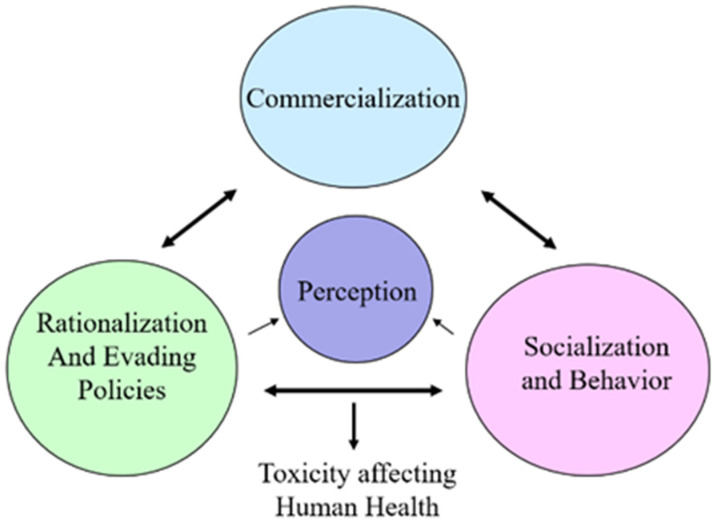
Oral nicotine products and marketing leading to evading perception and policies.

It may be surmised, based on the aspects of this concept by Ashforth and Anand, 2003 [29], as projected in Figure 1.

(1) Commercialization is the process by which unregulated policies are enacted as a matter of routine, often without conscious thought about their propriety or based on scientific knowledge;
(2) Rationalization, or evading policies, involves the process by which individuals who engage in usage accept socially constructed accounts that artificially legitimize the acts in their own eyes and perceptions;
(3) Socialization, or behavior changes, involve the process by which newcomers are thought to perform and accept the usage as a norm or alternative to smoking.

The problems created by the socially accepted illusion of perceived harmlessness toward nicotine products deserve significant consideration in future studies on ethical responsibility, organizational functioning, leadership accountability, and health concerns for these users. Researchers in toxicology and medicine continue to intellectually fight for better delivery of all information to these consumers so that all affected by this phenomenon can make more informed decisions about nicotine products’ social and bodily impacts on individuals and societal members. The untruth behind the use of illusory advertising to influence others into nicotine product consumption requires an undoing of its framework by regulatory agencies.

In order to examine the ONPs marketplace and enforce FDA, as well as local, flavor restrictions, we previously tried to classify and categorize snus and non-tobacco (synthetic) ONPs using the flavor wheel [27]. The current study is an effort to communicate several research gaps and address regulatory challenges for the usage of ONPs. The aim was to achieve the following goals: (1) expand and enhance the existing flavor wheel of snus and non-tobacco (synthetic), distributing them among the availability of flavors and various brands in the United States and Europe; (2) to encounter the availability of common flavors among snus and synthetic ONPs and, then, utilize use this semantic database to classify and identify various ONP flavors sold online; (3) given that both the ONP market and the regulatory environment have been rapidly progressing, our ONP flavor semantic database could be a very convenient tool to identify and classify flavors in the existing era, and it could also be useful for policymakers and researchers to survey the marketplace and check content for flavor restrictions. Thus, the findings of the present study could be utilized not only to notify potential policies but also to crystalize future research directions regarding ONPs flavors.

## 2. Materials and Methods

Utilizing the semantic database, we classified over 152 snus and 228 synthetic ONPs that we gathered from an online store. These ONPs were purchased from local vendors, and the ONPs belonging to European countries were purchased from an online store. Considering that each store may characterize or outline ONP flavors differently, we accessed the flavor information by employing different methods: (1) the source from which the flavor database is built includes Snusdirect, which is the website that provides access to consumers in North America and has the widest selection of products, (2) extracting and distributing the flavors of both snus and synthetic ONPs directly from the product website, which is presumably the most accurate, (3) extracting flavor categories in the brand website, (4) flavor descriptions in the product website, and (5) the consensus made between authors about flavors. Products are usually categorized on brand websites. Location distribution analysis for the brands producing ONPs is conducted by referencing the following two sources: (1) the brand website domain locations, which represent the target markets for their products; (2) company locations, which indicate the origin of the brands. Numerous companies utilize words that describe possible sensations and experiences for consumers to name their products, and judgment would be made based on the product description in such cases. Previously, we constructed two wheel diagrams, each consisting of one kind of product, constructed with flavors being color-coded, on which the flavor distribution analysis is done for synthetic and snus ONPs separately [27], and this is expanded in the current study.

## 3. Results

### 3.1. ONPs Distributors USA vs. Europe

**Figure 2:**
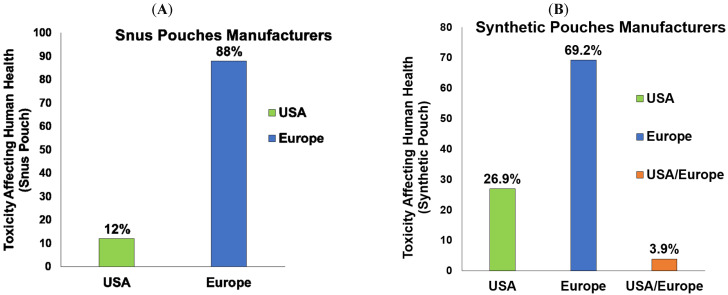
Frequencies of Snus and Synthetic ONP manufacturers USA vs. Europe: (**A**) Graphical distribution of Snus ONPs manufacturers in the United States vs. Europe. (**B**) Graphical distribution of Synthetic ONPs manufacturers in the United States vs. Europe.

First and foremost, we demonstrate the distributors of the snus ONPs and synthetic ONPs in the U.S. and European regions based on our collection of ONPs from local and online vendors. The European region carries a large share of about 88% of Snus ONP distributors as compared to the United States, which consists of 12% of distributors, as shown in Figure 2A. Moreover, the distributors of synthetic ONPs are about 26.9% in the United States, 69.23% are in Europe, and 3.84% are common in the United States/Europe, as shown in Figure 2B.

### 3.2. Distribution of ONPs on Basis of Their Individual Brands Snus vs. Synthetic Nicotine

We classified the brands marketing snus and synthetic ONPs and were able to via the online ONPs dealers and our samples. We were able to encounter 25 brands marketing snus ONPS and 28 brands marketing synthetic ONPs. The frequency counts of snus ONP brands were Camel (7.2%), Catch (2.0%), Copenhagen (7.2%), Ethan (2%), General (3.3%), Granit (2.6%), Grizzly (7.2%), Grovsnus (2.6%), Jakobsson (5.9%), Kaliber (3.3%), Kapten (3.9%), Knox (5.3%), Kodiak (1.3%), Kronan (3.3%), Lab (3.3%), LD (2.6%), Longhorn (3.3%), Lundgrens (7.2%), Mustang (0.7%), Oden (5.3%), Offroad (2.0%), Rite (2.0%), Roda Lacket (0.7%), Skoal (13.8%), and Smalands Broakssnus (2.0%), as represented in Figure 2A.

**Figure 3:**
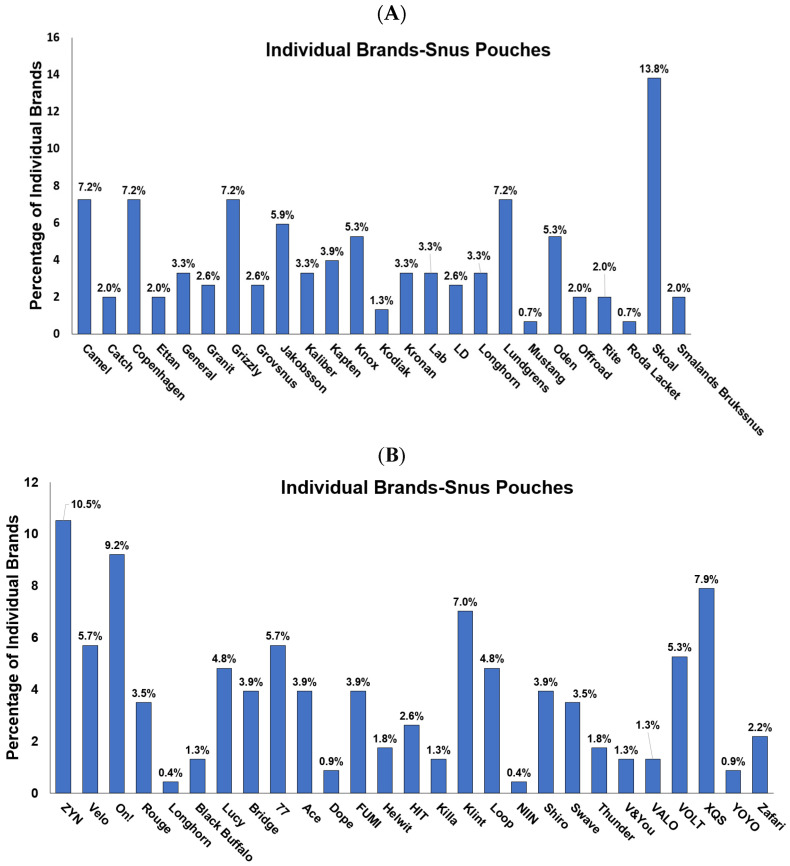
Individual Distribution of ONPs based on the various brands that sell them: (**A**) Graphical representation of the Frequencies of Snus ONP Brands *n* = 152; (**B**) Graphical representation of the Frequencies of Synthetic ONP Brands *n* = 228.

The frequency counts of synthetic ONP brands were as follows: ZYN (10.5%), Velo (5.7%), On! (9.2%), Rouge (3.5%), Longhorn (0.4%), Black Buffalo (1.3%), Lucy (4.8%), Bridge (3.9%), 77 (5.7%), Ace (3.9%), Dope (0.9%), Fumi (3.9%), Helwit (1.8%), HIT (2.6%), Kills (1.3%), Klint (7.0%), Loop (4.8%), NIIN (0.4%), Shiro (3.9%), Swave (3.5%), Thunder (1.8%), V&You (1.3%), Valo (1.3%), Volt (5.3%), XQS (7.9%) Yoyo (0.9%), and Zafari (2.2%), as represented in Figure 2B. Skoal, Camel, Copenhagen, Grizzly, and Lundgrens were the top marketed brands of snus ONPs; on other hand, the top brands from which synthetic ONPs are marketed are ZYN, On!, XQS, and Klint (Figure 3A,B).

### 3.3. Availability and Distribution of Flavors Snus vs. Synthetic Nicotine

**Figure 4:**
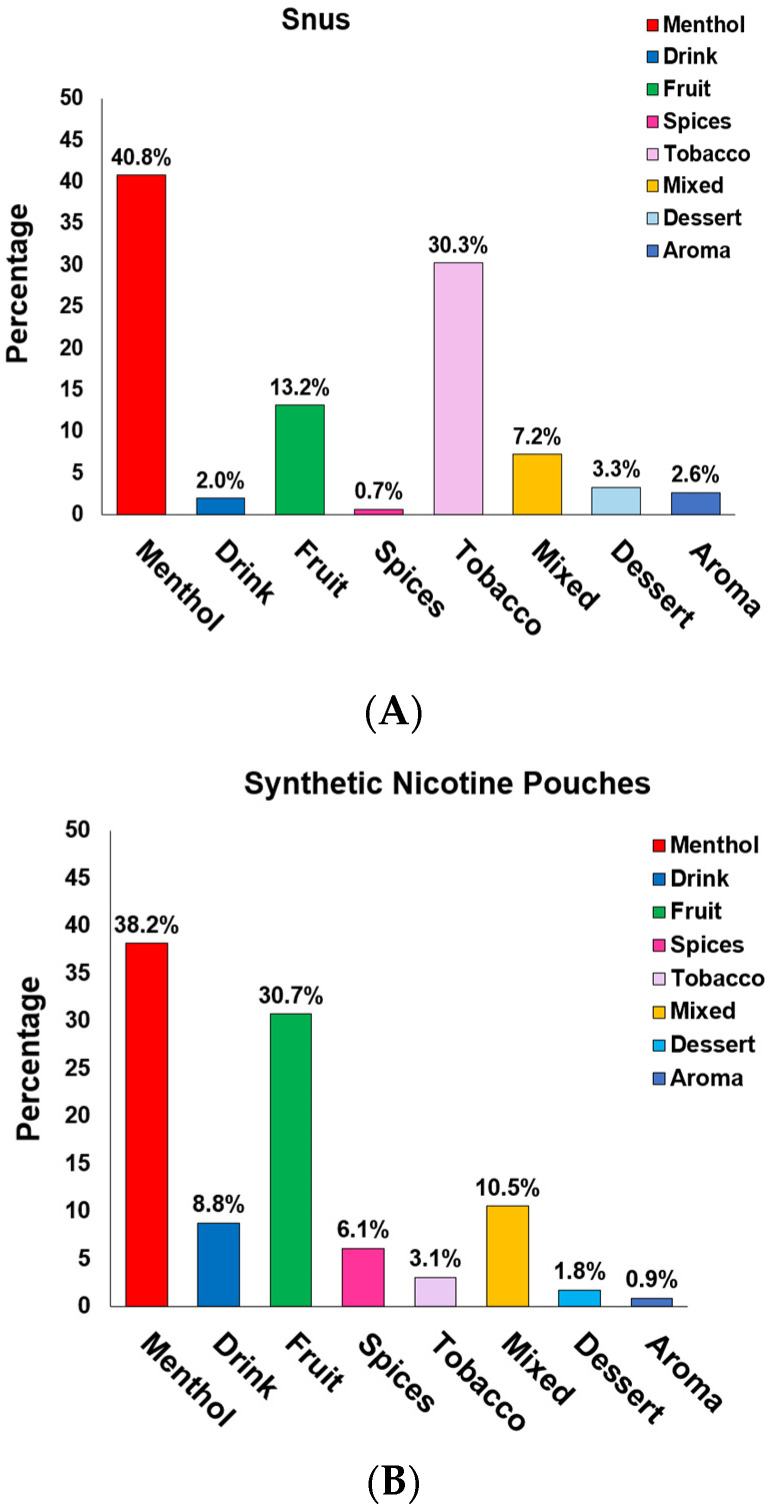
Flavor distribution Snus vs. Synthetic oral ONPs: (**A**) Frequencies of Snus ONPs Containing Certain Flavors *n* = 228. (**B**) Frequencies of Synthetic ONPs Containing Certain Flavors *n* = 152, comparing the common flavor distribution of snus vs. synthetic ONPs.

In Figure 4A,B, we present a frequency plot of the 228 snus ONPs and 152 synthetic ONPs in our sample by classifying their flavor description into one of the following: (1) tobacco flavor, i.e., the product contains natural tobacco (30.3%) in the case of snus ONP flavors such as Natural, Strong, Original, Straight, Classic Original, and Extra Strong. For synthetic ONPs the tobacco flavor frequency is (3.1%), and the flavors are usually Smooth, Original, and Straight; (2) menthol flavors only, i.e., the product contains mint, wintergreen, spearmint, cool mint. eucalyptus, classic mint, smooth mint, and frosted flavors for snus ONPs 40.8%, and synthetic ONPs 38.2% include menthol flavors such as cold mint, iced mint, soft mint, peppermint, and many more; (3) fruity flavor, which means the product contains one or more fruity flavors such as citrus, berry, dragon fruit, mango, and no supplemented flavor from any of the other principal categories; this includes 13.2% fruit flavors for snus ONPs and 30.7% synthetic ONPs; (4) dessert/ candy/other sugary flavors, where snus ONPs have 3.2% and synthetic ONPs have 1.8% dessert flavors. (5) Aroma flavors for snus are 2.6%, and for synthetic pouches, they are 0.9%. There are also (6) spice flavors such as cinnamon, (7) drink flavors such as coffee, (8) other flavors, namely Raspberry/Liquorice and Original Portion, which were classified as mixed flavors; their frequency for snus ONPs is 7.2%, and synthetic ONPs is 10.5%. Menthol and tobacco were the most prevalent flavors among snus ONPs; on the other hand, Menthol and Fruit flavors were the most prevalent flavors among synthetic ONPs.

Based on the availability of ONP flavors, we next sought to perceive common flavors available in each category, such as tobacco, menthol, fruit, dessert, spice, aroma, and the one classified as mixed flavors, in the case of the snus and synthetic ONPs based on of online shops and our samples.

**Figure 5:**
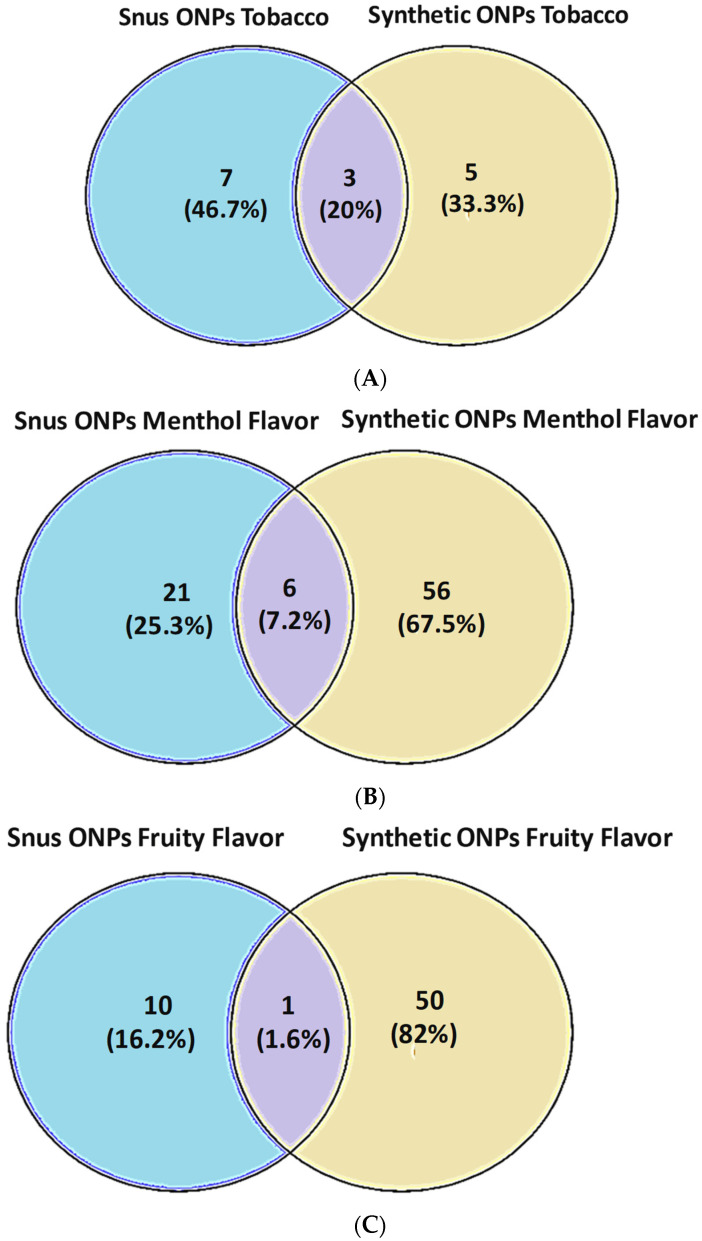
Comparison of available flavors of Snus vs. Synthetic ONPs: (**A**) Venn diagram indicating the common tobacco flavors marketed among snus and synthetic ONPs; (**B**) Venn diagram indicating the common menthol flavors marketed among snus and synthetic nicotine containing ONPs. (**C**) Venn diagram (using software Venny 2.0.2) indicating the common Fruit flavors marketed among snus and synthetic nicotine containing ONPs.

Among tobacco flavors, the frequency of common flavors was 20% in snus vs. synthetic ONPs (Figure 5A).

Among menthol flavors, about 7.2% are common among snus vs. synthetic ONPs (Figure 5B).

In the category fruit flavors—which is the most diverse category—interestingly, only about 1.6% of the common fruit flavors are available among snus vs. synthetic ONPs (Figure 5C), which indicates that synthetic ONPs have attractive fruit-flavored pouches available unlike snus pouches.

No common flavors were available in the aroma, spices, and mixed flavor ONPs category.

### 3.4. Application of ONPs in Research Studies

Within a minimal time, ONPs have emerged in the research field. Various research studies and case reports using ONPs in various perspectives have been published. The name does imply oral pouches, but it does tend to affect the other human organs (systemically) as well. Moreover, studies demonstrated that the regular utilization of smokeless nicotine products is related to a higher risk for diseases such as cancers, Parkinson’s disease, birth defects, oral submucosal fibrosis, periodontal diseases, cardiovascular disease, and type 2 diabetes [27,28]. As both snus and ONPs are not directly inhaled through the lungs, the flavoring chemicals, nicotine, and the byproducts within these products might be secreted across the membrane of the buccal cavity into the systemic circulation; these byproducts can then act locally on various tissues within the human body; responses of these are associated with the cardiopulmonary system via kidneys, liver, microvasculature, esophagus, and the pancreas [35,36,37]. Although these products are shown to have their toxic effects, the number of pouches used per day for a number of months/years (as with smokers with cigarettes pack/year or puffs/day for vapers) required to have potential periodontal effects are not yet clinically standardized. These commercial products may contain varying concentrations of nicotine, from 3 mg to 32 mg per pouch, with other flavoring agents, including triacetin, benzyl alcohol, menthol, and cooling agent WS-23. Studies also depict the high possibility of the absorption of byproducts/chemicals of this oral smokeless product to merge with the lung microvasculature along the airways [27]. An in vitro study investigated the oral nicotine pouch products in terms of oral irritation in the EpiGingival™ 3D tissue model and artificial saliva. This study reported oral pouches as non-genotoxic, non-cytotoxic, and non-mutagenic [38]. An interesting study by Dawler et al. studied the diversity among oral tobacco products and reported the ability of these products to cause a high range of carcinogenic effects to users due to the presence of nicotine and tobacco-specific nitrosamines (TSNAs) [39]. Concerning the additional approaches of the ONPs, studies also report the impact of ONPs on lungs as case studies put forward that pulmonary aspirations of smokeless tobacco products activate recurrent pulmonary infiltrations and multifocal airway obstructions in the lungs of the users, probably causing aspiration pneumonia [40]. Recent evidence investigated the pharmacokinetic parameters of ONPs and reported that oral nicotine pouches carry the potential to be an acceptable substitute for adult smokers, as the users can achieve adequate nicotine levels to deliver into the body [41]. Our recent in vitro investigation focused on oral–pulmonary health effects of snus and ONPs, indicating that the flavored ONPs are risky and likely to cause local and systematic toxicological responses during chronic consumption [27].

### 3.5. Potential Molecular Targets Due to Exposure to Oral Nicotine Products Triggering Possible Signaling Cascades

One of the prime components of cigarette smoking is nicotine, which can modulate cell proliferation and trigger apoptosis both in normal cells as well as various human cancer cell lines derived from several organs [42]. Previously, various research studies disclosed the involvement of nicotine in activating several signaling cascades. Yuge et al., 2015, demonstrated that nicotine exposure decreased the reduction in T24 cells via elevating pAkt and pS6 expressions in vitro and in vivo via stimulating the PI3K/Akt/mTOR signaling in bladder cancer [43]. Another study showed that the involvement of nicotine in the progression and development of colon cancer is responsible for cell proliferation regulation and the suppression of apoptosis [43]. Evidence also reports that we found that nicotine stimulates the levels of apoptotic markers, such as cleaved caspase-3, via increasing oxidative stress and enhancing the number of apoptotic cells upon podocyte injury [44]. Nicotine in e-cigarettes also activates the EMT process, causing lung cancer [45].

**Figure 6:**
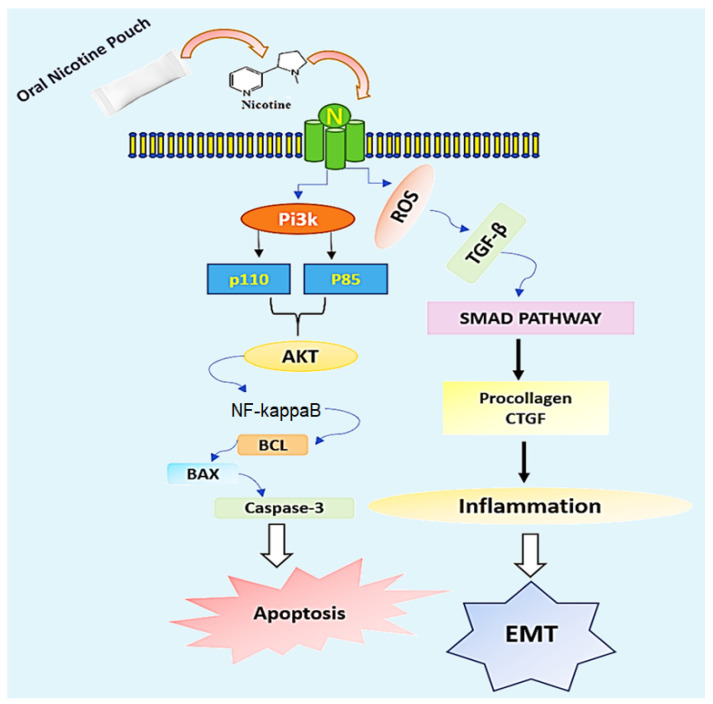
Schematic Depicting Possible Modulation of Signaling Cascades Activation by Oral Nicotine Pouches-Mediated Chemical Moieties.

With regards to our previous findings of ONPs interacting and activating signaling molecules, here, we predict interesting interactions of ONPs by studying their role in targeting the apoptosis and epithelial–mesenchymal transition signaling cascades. Nicotine in ONPs could possibly engage the class-Ia PI3K, which stands for heterodimer constructed of the p110 catalytic and p85 regulatory subunits. This further leads to the recruitment AKT and NF-kB, further activating the apoptotic proteins Bcl, Bax, and Caspase-3, triggering the apoptosis process. Additionally, ONPs elevate the Reactive Oxygen Species, ROS [27], which can stimulate the levels of TGF-β1, triggering the activation of SMAD pathway via modulating the procollagen CTGF, augmenting inflammation, and ultimately activating the process of EMT (Figure 6).

Recent literature indicates the involvement of menthol flavor in e-cigarette in upregulating the cytosolic calcium, thereby stimulating the TRPV1 receptor and leading to the activation of certain kinases and cytokines. [46]. With reference to this, we presume the involvement of menthol-flavored ONPs in modulating TRPV1 receptors via increasing the cytosolic calcium might be responsible for the recruitment of cytokines and kinases.

## 4. Discussion

The present study provides a timely and instructive insight that could be helpful for researchers working on ONPs to classify and analyze available flavors of ONP sold locally and online, which can be marked with various composite flavor profiles such as menthol + fruit, dessert + fruit, fruit + aroma, and spice + fruit, given that these fascinating flavors are most likely the major attributes to attract young and adolescents to try ONPs [13,22,23]. Here, we expand the existing ONP flavor wheel of snus (natural) and the non-tobacco (synthetic) flavor wheel, dividing them among the availability of their flavors and discrete brands marketing ONP in the United States and Europe. ONPs are marketed in the United States and Europe as a substitute for tobacco smoking [1,2,3]. The current review also explains the social behavioral aspects of ONPs. We could review that the European region demonstrates a large share of about 88% of snus ONP distributors, whereas the United States contains about 12% of snus distributors, while synthetic ONPs display 26.9% of distributors in the United States, 69.23% in Europe, and 3.84% combined in the United States/Europe. Studies report Velo, On!, and ZYN as the most emphasized brands of ONPs [47]. Here, we attempted to analyze, from our sample ONPs, the most marketed brands of the categories of ONPs. Our analysis evaluating the top marketed U.S. and Europe brands of ONPs implied Skoal, Camel, Copenhagen, Grizzly, and Lundgrens as the top marketed snus ONPs, and the top brands marketing synthetic ONPs were ZYN, On!, XQS, and Klint. In 2020, it was reported that the most commonly sold flavor group of ONPs were mint flavors (including mint, wintergreens, and spearmint), followed by fruit flavors, cinnamon flavors, and coffee flavors [48]. In agreement with this, our analysis found that menthol, along with tobacco, was the most prominent flavors among snus or natural ONPs, and menthol, along with fruity flavors, was the most dominant flavor in the synthetic ONPs category. This includes various flavoring chemicals, including triacetin, benzyl alcohol, menthol, and cooling agent WS-23. Next, we sought to encounter what could be the possibility of common flavors available among both snus and synthetic ONPs. Interestingly, about 20% of common flavors are available among tobacco in snus versus synthetic ONPs, 7.4% are available in menthol, and the least are the fruit, which depicted a frequency of 1.6% in snus versus synthetic ONPs.

ONPs have also shown lower cellular toxicity in vitro in human bronchial epithelial cells (H292), human oral fibroblasts (HGF), human lung epithelial cells (BEAS-2B), and human liver epithelial cells (HepG2) [27,49,50]. Moreover, lower mutagenicity was found for ONPs in *Salmonella typhimurium*, and lower genotoxicity was found in V79 hamster lung cells [50]. It is also shown that ONPs have lower pharmacokinetics and addictive potential compared to traditional tobacco products [51]. Although thought to have lower cytotoxicity, ONPs can still cause injuries in cells and trigger inflammatory responses. We previously reported higher levels of lactate dehydrogenase (LDH), reactive oxygen species (ROS), and inflammatory cytokines (TNF-α, IL-6, and IL-8) in human gingival epithelial cells (HGEPp), human lung epithelial cells (BEAS-2B), and human bronchial epithelial cells (16-HBE) after treatment with ONPs [27]. Studies have also indicated an association between ONPs usage and risks for various diseases, including Parkinson’s disease, cancer, birth defects, type II diabetes, oral submucosal fibrosis, periodontal diseases, and cardiovascular diseases [39,43]. Here, we also present an insight into the research perspectives of ONPs based on perception, behavior, and toxicology. Reporting that ONPs can also participate in modulating signaling cascades via enhancing apoptosis and EMT process, present findings could be useful for putative notification policies, as well as for identifying future research approaches for flavored ONPs.

## 5. Future Perspectives

For better understanding and to be up to date with a variety of emerging available ONP flavors in the local and online markets, the socio-economical aspect, behavior, chemistry, toxicity/harmful effects, and creativity in data sciences should be applied urgently, especially for classifying and identifying ONP flavors/flavoring chemicals, nicotine strengths, and other descriptions that would be important in conducting surveillance at ONP brand websites, as well as social media. Monitoring ONP flavor profiles, characteristics, and toxicities should be implied, as it might be critical for recognition of the comparative nicotine and tobacco product appeals in the marketplace.

## 6. Conclusions

Several new flavored ONPs are currently popular among local vendors, as well in the online market. Tobacco, menthol, and fruit-flavored ONPs could be alternatives of tobacco smoking for cigarette smokers. Considering the ONP products’ marketing, with numerous flavor profiles and the majority of these oral products consisting of tobacco/menthol/fruit flavor, it is likely to impact regulation and marketing disclaimers on these fewer products. The flavor is an important attribute of oral nicotine products, especially for young users. The development of new ONP flavors could be related to the initiation and advances of usage of ONPs among youth and adults in the United States and Europe. Regulations on ONP flavors are under the authority of the U.S. Food Drug Administration. The current review can be used to notify the putative policies, as well as to shed light on future aspects of research directions regarding ONPs flavors. Further, it would be logical to decide how the merchandise responds in terms of compliance and non-compliance with flavor restrictions by the regulatory agencies.

## Data Availability

We declare that we have provided all the data in figures.
